# Inefficient Complement System Clearance of *Trypanosoma cruzi* Metacyclic Trypomastigotes Enables Resistant Strains to Invade Eukaryotic Cells

**DOI:** 10.1371/journal.pone.0009721

**Published:** 2010-03-16

**Authors:** Igor Cestari, Marcel I. Ramirez

**Affiliations:** Laboratório de Biologia Molecular de Parasitas e Vetores, Instituto Oswaldo Cruz - Fiocruz, Rio de Janeiro, Brazil; Agency for Science, Technology and Research (A*STAR), Singapore

## Abstract

The complement system is the main arm of the vertebrate innate immune system against pathogen infection. For the protozoan *Trypanosoma cruzi*, the causative agent of Chagas disease, subverting the complement system and invading the host cells is crucial to succeed in infection. However, little attention has focused on whether the complement system can effectively control *T. cruzi* infection. To address this question, we decided to analyse: 1) which complement pathways are activated by *T. cruzi* using strains isolated from different hosts, 2) the capacity of these strains to resist the complement-mediated killing at nearly physiological conditions, and 3) whether the complement system could limit or control *T. cruzi* invasion of eukaryotic cells. The complement activating molecules C1q, C3, mannan-binding lectin and ficolins bound to all strains analysed; however, C3b and C4b deposition assays revealed that *T. cruzi* activates mainly the lectin and alternative complement pathways in non-immune human serum. Strikingly, we detected that metacyclic trypomastigotes of some *T. cruzi* strains were highly susceptible to complement-mediated killing in non-immune serum, while other strains were resistant. Furthermore, the rate of parasite invasion in eukaryotic cells was decreased by non-immune serum. Altogether, these results establish that the complement system recognizes *T. cruzi* metacyclic trypomastigotes, resulting in killing of susceptible strains. The complement system, therefore, acts as a physiological barrier which resistant strains have to evade for successful host infection.

## Introduction

Chagas disease is an illness that affects 18 million people in Latin America [1]. It is caused by the protozoan parasite *Trypanosoma cruzi* and is mainly transmitted by triatomine insects, although congenital, oral and blood transfusion transmission has also been reported [2], [3], [4], [5], [6], [7]. In the insect vector, *T. cruzi* are present as replicative non-infective epimastigotes, which differentiate to non-replicative infective metacyclic trypomastigotes. During the infection, metacyclic trypomastigotes have to evade the host innate immune system and infect cells to progress in the infection. Parasites that succeed in infecting host cells differentiate to amastigotes, an intracellular replicative stage, which after several divisions differentiate to bloodstream trypomastigotes that disrupt cells, infecting new cells, or are taken by insect vectors restarting the life cycle [8].

The first barrier against a microbe's infection of vertebrates is the innate immune system, encompassing the complement system and phagocytic and natural killer cells. The complement system is one of the most effective innate mechanisms of host defense against a pathogen infection. It is composed of several proteins activated in a cascade that culminate with membrane attack complex formation on the pathogen surface, causing killing by cell lysis [9]. The complement system can be activated by three pathways: classical, activated when IgG or IgM bind to the pathogen surface and associate with the C1 complex (C1qr_2_s_2_), which cleaves C2 and C4 to form C3 convertase (C4b2a); lectin, activated when mannan-binding lectins (MBLs), L-ficolins or H-ficolins bind to the pathogen surface and associate with MBL-associated serine protease-2 (MASP2) to cleave C2 and C4, generating C3 convertase (as in the classical pathway); and alternative, activated when C3b binds to the pathogen surface and associates with factor B to form C3 convertase (C3bBb) [9].

During *T. cruzi* infection, it is estimated that 10^4^ to 10^5^ metacyclic trypomastigotes are released by the insect vector [10], [11], which rapidly penetrate the host skin [12], [13]. In the absence of specific antibodies, the complement alternative and lectin pathways are responsible for triggering the complement system against the pathogens in the bloodstream. Metacyclic trypomastigotes, derived from triatomine vectors or axenic cultures, were shown to be killed by the complement with non-immune sera [14], [15]. Also, C3b was detected on the surface of metacyclic trypomastigotes, although less efficiently than on epimastigotes [16], [17]. Gp72 was identified as the main C3b acceptor in epimastigotes, but it is a poor C3b acceptor in metacyclic trypomastigotes [16]. The reduced activation of the alternative pathway by metacyclic trypomastigotes compared to epimastigotes is thought to be due to the failure of factor B to interact with the surface deposited C3b [18]. On the other hand, a rapid binding of MBLs, L-ficolins and H-ficolins on the surface of metacyclic trypomastigotes has been shown, providing evidence that the lectin pathway is activated in non-immune serum by *T. cruzi* [19]. Furthermore, bloodstream trypomastigotes sensitized with antibodies from acutely infected animals were killed by the complement system in human serum [20], [21], and anti-α-galactosyl antibodies recognizing O-linked oligosaccharides on *T. cruzi* mucin-like GPI-anchored proteins were identified in chronic phase Chagas disease patients [22], [23]. Altogether, these data support the idea that the complement system can recognize and kill the infectious stages of *T. cruzi*.

The expression of complement system inhibitors, such as complement C2 receptor inhibitor trispanning, complement regulatory protein, calreticulin and decay accelerating factor, has been demonstrated in *T. cruzi*; these molecules help *T. cruzi* to avoid complement killing [24], [25], [26], [27]. However, the levels of expression and functionality of these molecules could be different among *T. cruzi* strains, since resistance to complement killing has been demonstrated to vary among them [20], [24]. Despite the mechanisms of resistance to the complement system, the differences of *T. cruzi* strains and the high parasite load required for infection suggest that not all metacyclic trypomastigotes succeed in infecting the host [12], [28], [29], [30], [31].

Aiming to understand how *T. cruzi* metacyclic trypomastigotes interact with the human complement system, we analysed the effect of the complement system on the lysis and eukaryotic cell invasion by several *T. cruzi* strains. Firstly, through C3b and C4b deposition assays we found that *T. cruzi* mainly activate the lectin and alternative pathways in non-immune serum. *In vitro* assays revealed that metacyclic trypomastigotes of several strains of *T. cruzi* differ in their capacity to resist the complement system, and some of them were killed after incubation in non-immune human serum. Moreover, the rate of *T. cruzi* invasion of eukaryotic cells was significantly reduced in the presence of non-immune human serum, showing that the complement system acts as a physiological barrier for *T. cruzi* infection and strains resistant to complement system have a higher chance to succeed in the infection.

## Results

### 1- Complement activating factors bind to the surface of *T. cruzi* strains

To determine whether different strains of *T. cruzi* would activate the complement system in human serum, the binding of complement activating factors to these strains was analyzed. *T. cruzi* epimastigotes were used because they are sensitive to complement killing; therefore, they activate the complement system. Strains from congenitally transmitted Chagas disease patients, in addition to recently isolated strains from primate, opossum and rodent were studied (Table 1). The strains Y, Silvio X10/6, and CL Brener are used by several laboratories and were also included in some of the experiments.

**Table 1:** *Trypanosoma cruzi* strains, hosts and biome.

Phylogenetic group/Isolate	Host	Biome
TCI 8612	*Rattus rattus* (rodent)	Piauí/Brazil
TCI 8628	*Rattus rattus* (rodent)	Jaguaruana, Ceará/Brazil
TCI Gambá 05	*Didelphis aurita* (opossum)	Poço das Antas Reserve, Rio de Janeiro/Brazil
TCII 750	*Leontopithecus rosalia* (primate)	Poço das Antas Reserve, Rio de Janeiro/Brazil
TCII 812	*Leontopithecus rosalia* (primate)	Poço das Antas Reserve, Rio de Janeiro/Brazil
TCII 840	*Leontopithecus rosalia* (primate)	Poço das Antas Reserve, Rio de Janeiro/Brazil
TCII 860	*Leontopithecus rosalia* (primate)	Poço das Antas Reserve, Rio de Janeiro/Brazil
TCII R4	*Triatomae brasilienses* (triatominae)	João Costa, Piauí/Brazil
TCII MLCD88	*Leontopithecus chrysomelas* (primate)	REBIO-Una, Bahia/Brazil
TCII M3	*Didelphis albiventris* (opossum)	White scrub, Piauí/Brazil
CL Brener	*Triatoma infestans* (triatominae)	Rio Grande do Sul/Brazil
Silvio X10/6	human acute infection	Brazil
Tapia	Human (newborn child)	Molinos, Salta, Argentina
Gareca	Human (14 months child)	Anta/Argentina
Mendez	Human (newborn child)	Campo Quijano, Salta/Argentina
Y	human acute infection	São Paulo/Brazil

**Figure 1:**
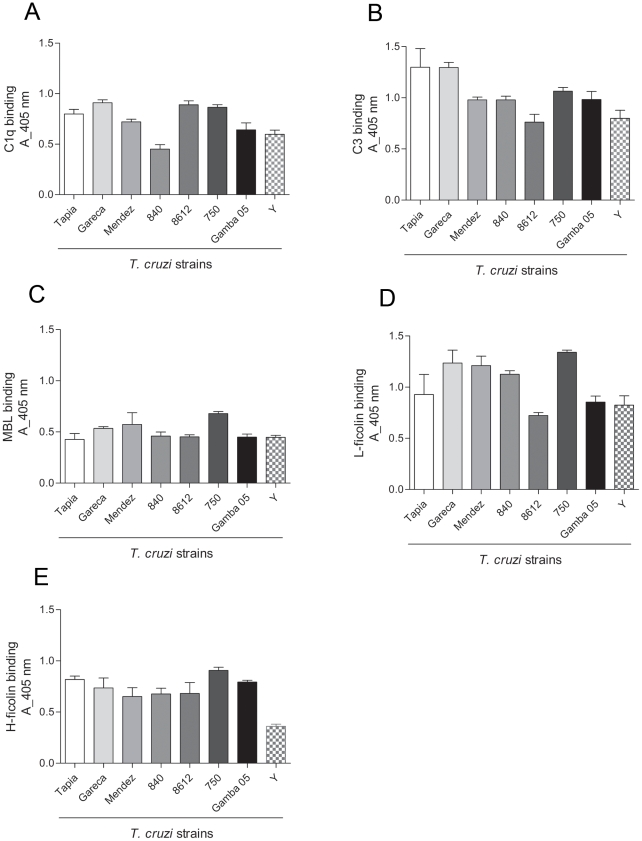
Complement activating factors bind to *T. cruzi* surface. A) ELISA for detection of C1q bound to *T. cruzi* epimastigotes of several strains. Parasites adsorbed in ELISA plates were incubated with 5% NHS for 1 h at 37°C. Anti-C1q antiserum was used for detection and reactions were developed with ABTS-peroxidase solution measured at 405 nm. B) Detection of C3b bound to *T. cruzi*. C) Detection of MBL bound to *T. cruzi*. D) Detection of L-ficolins bound to *T. cruzi*. E) Detection of H-ficolins bound to *T. cruzi*. Experiments in B, D and E were performed as in A, except that for detection were used antiserum against C3, L-ficolins and H-ficolins, respectively. Experiments in C were performed as in A, except that 20% NHS and anti-MBL antiserum were used.

Binding of C1q, involved in classical pathway activation; MBLs, L-ficolins and H-ficolins, for the lectin pathway, and C3 for the alternative pathway were analyzed by ELISA. All these molecules were detected bound to the parasites after incubation with non-immune human serum (Figure 1A to E). Although a slight difference in the binding was detected among the strains, the results show that all *T. cruzi* strains are recognized by the complement activating molecules.

### 2- *T. cruzi* strains activate mainly the lectin and alternative complement pathways

**Figure 2:**
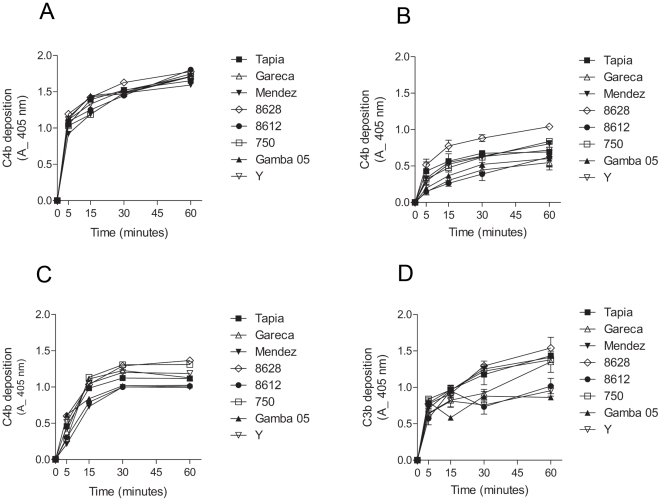
*T. cruzi* activate mainly the lectin and alternative complement pathways. A) C4b deposition on *T. cruzi* epimastigotes of several strains. Parasites adsorbed in ELISA plates were incubated in 5% NHS from 5 to 60 minutes at 37°C, followed by anti-C4 antiserum incubation. Reactions were developed with ABTS-peroxidase solution measured at 405 nm. B) C4b deposition in epimastigotes of *T. cruzi*. Experiments were performed as in A, except that MBLs/L-ficolins-depleted sera were used. C) C4b deposition in epimastigotes of *T. cruzi*. Experiments were performed as in A, except that C1q-depleted sera were used. D) C3b deposition in *T. cruzi* epimastigotes. Experiments were performed as in A; except that sera treated with 10 mM EGTA and 7 mM MgCl_2_ were used. Detection was made with anti-C3 antiserum.

To determine the contribution of each pathway to the complement activation by *T. cruzi* strains, kinetic assays were performed to detect C3b and C4b deposition on the parasite surface. To analyze the classical and lectin pathway contribution, C4b deposition assays were performed with normal human serum (classical and lectin pathway activation), with serum depleted of MBLs and L-ficolins (mainly classical pathway activation) and with serum depleted of C1q (lectin pathway activation). C4b deposition in normal human serum was rapid and similar between all strains, reaching 50% (absorbance  = 1.0) in less than 5 minutes (Figure 2A). However, C4b deposition in MBLs/ficolins-depleted serum was slower than in normal human serum (Figure 2B). C4b deposition in MBLs/ficolins-depleted serum varied among the strains. Between 2 to 4 times less deposited C4b was detected in assays with MBLs/ficolins-depleted serum than with normal human serum, even after 1 hour of incubation. To examine the ability of the lectin pathway to recognize *T. cruzi* and activate the complement, the C4b deposition was performed in serum depleted of C1q. The velocity of C4b deposition in this serum was slightly slower than in normal human serum, but higher than in serum depleted of MBLs/ficolins (Figure 2C). Substantial depletion of MBLs/L-ficolins and C1q from sera was confirmed by ELISA (Figure S1). These results indicate that *T. cruzi* activate both classical and lectin pathways; although, as observed before [19], the lectin pathway seems to be more effective than the classical pathway in recognizing *T. cruzi* in non-immune serum. To verify if these strains activate the alternative pathway, C3b deposition was analyzed in serum treated with EGTA to chelate Ca^2+^. Importantly, the classical and lectin pathways are Ca^2+^- and Mg^2+^-dependent, whereas the alternative pathway is only Mg^2+^-dependent [9]. C3b deposition increased gradually through time for all strains (Figure 2D). It is noteworthy that strains 8612, Y and Gamba 05 activated the alternative pathway less than the other strains analyzed. Together, these results indicate that upon contact with human serum, *T. cruzi* strains activate mainly the lectin and alternative pathways of the complement system.

### 3- *T. cruzi* metacyclic trypomastigotes are killed by the complement system

**Figure 3:**
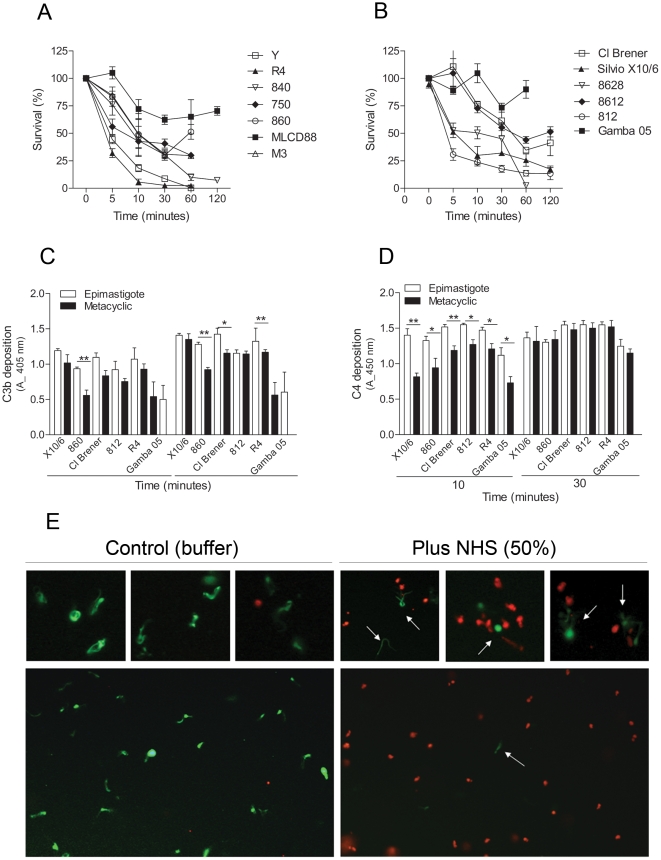
*T. cruzi* metacyclic trypomastigotes are killed by the complement system. A and B) Kinetics of complement-mediated killing assays with metacyclic trypomastigotes of several *T. cruzi* strains. Parasites were incubated with 50% NHS at 37°C for 5 to 120 minutes and survivors were counted. C) C3b deposition in metacyclic trypomastigotes of several *T. cruzi* strains. Parasites adsorbed on ELISA plates were incubated in 5% sera treated with 10 mM EGTA and 7 mM MgCl_2_ for 10 and 30 minutes at 37°C, followed by anti-C3 antiserum incubation. Reactions were developed with ABTS-peroxidase solution measured at 405 nm. D) C4b deposition in metacyclic trypomastigotes of several *T. cruzi* strains. Experiments were performed as in C, except that 5% NHS anti-C4 antiserum was used. E) Live/dead viability fluorescence assay. Metacyclic trypomastigotes (812 strain) were incubated in 50% non-immune human serum for 30 minutes at 37°C (plus NHS), or in PBS as control (buffer). After that, parasites were incubated with 2 µM of calcein-AM and 4 µM of ethidium homodimer-1 for 30 minutes at RT, and slides were analysed by fluorescence microscopy. Calcein-AM dye is retained by live cells and produces green fluorescence after activation by cytoplasmic esterase, whereas ethidium homodimer-1 is excluded by the plasma membrane of live cells, enters cells with damaged membranes, and produces red fluorescence upon binding to nucleic acids in dead cells. * *p*<0.05; ** *p*<0.01.

We have seen previously that the majority of epimastigotes of *T. cruzi* strains are susceptible to complement killing in normal human serum at nearly physiological conditions (50% of serum at 37°C) after incubations ranging from 5 to 30 minutes (not shown). We decided to analyze whether the metacyclic trypomastigotes of *T. cruzi* strains would be resistant to complement killing. Strikingly, some *T. cruzi* strains were killed by human complement at nearly physiological serum concentration, 50% (Figure 3 A and B). Parasites from the strains Y, R4 and 8628 were completely killed between 30 to 60 minutes of incubation, whereas the survival rate of the strains Silvio X10/6, 840, 750, M3 and 812 were no more than 30% after a 60 minute incubation. The survival rate of the strains CL Brener, 860 and 8612 was about 50%; and higher survivals were detected for strains MLCD88 and Gamba 05, about 75%. We also did not find any correlation between the complement killing susceptibility of the strains and their respective phylogenetic groups. It is noteworthy that the purity of metacyclic trypomastigotes used in these assays was always above 95%, as confirmed by morphological parameters, and they also expressed the antigen-specific gp82, detected by Western blotting (exemplified in Figure S2). *T. cruzi* is refractory to the African trypanosome lytic factor apolipoprotein-I [32], [33], [34], excluding the possibility that this factor mediates the lysis. Furthermore, inhibition of the complement cascade by chelating the Ca^2+^ and Mg^2+^ from the serum with EDTA, treatment of the serum with protease inhibitors or the use of a complement consumed serum failed to lyse *T. cruzi* metacyclic trypomastigotes (Figure S3). To obtain further evidence that metacyclic trypomastigotes are killed by activation of the complement system, we decided to compare the deposition of C3b and C4b between epimastigotes and metacyclic trypomastigotes. There was a higher deposition of C3b and C4b on epimastigotes than on metacyclic trypomastigotes at 10 minutes incubation with non-immune serum (Figure 3C and D). However, by 30 minutes no significant difference for C4b deposition on epimastigotes and metacyclic trypomastigotes was detected (Figure 3D), confirming that metacyclic trypomastigotes activate the complement system.

Using a live/dead cells fluorescence-based assay we confirmed that metacyclic trypomastigotes were being killed after human serum incubation. For this assay, metacyclic trypomastigotes of the strain 812 were incubated with or without non-immune human serum at concentration of 50% for 30 minutes at 37°C, followed by labeling with calcein-AM (green, to detect live cells) and ethidium homodimer-1 (red, for dead cells). Serum incubation resulted in almost complete parasite killing (red), while non-treated parasites were alive (green) and motile. There were few parasites survivors after serum treatment, as can be observed in Figure 3E (arrows). Together, these results show that *T. cruzi* metacyclic trypomastigotes activate the complement system, and there are strains sensitive and resistant to complement-mediated killing by human serum.

### 4- *T. cruzi* invasion of eukaryotic cells is limited by the complement system

After showing that different *T. cruzi* strains vary in their ability to resist the complement system, and that some strains are susceptible to complement killing, we decide to investigate how efficiently the complement system can control metacyclic trypomastigotes invasion of eukaryotic cells in an *in vitro* assay that simulates physiological conditions.

**Figure 4:**
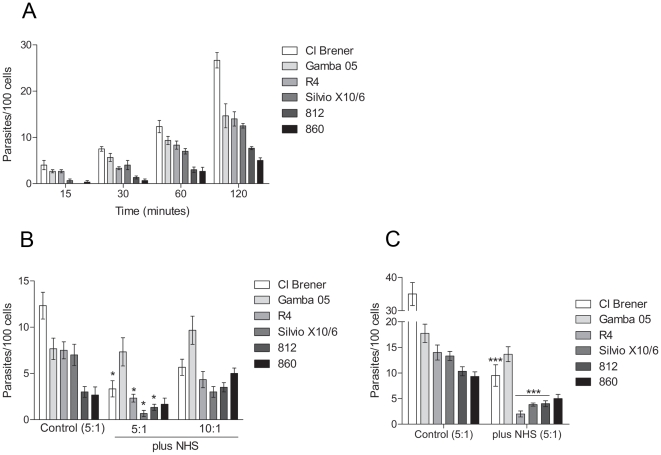
*T. cruzi* invasion in eukaryotic cells is limited by the complement system. A) Kinetics invasion of Vero cells by *T. cruzi* metacyclic trypomastigotes. Parasites (5×10^5^) were incubated with Vero cells (1.0×10^5^, ratio 5∶1, parasites:cells) for 15 to 120 minutes at 37°C, and intracellular parasites in at least 500 cells were quantified. B) Invasion assays of Vero cells by metacyclic trypomastigotes of *T. cruzi* strains with or without NHS. Parasites (5×10^5^ or 1.0×10^6^, ratios 5∶1 and 10∶1, parasites:cells) were incubated with Vero cells (1.0×10^5^) in 50% NHS for 60 minutes at 37°C. As controls, parasites and cells (ratio 5∶1) were incubated without NHS. Intracellular parasites in at least 500 cells were quantified. C) Invasion assays of Vero cells by metacyclic trypomastigotes of *T. cruzi* strains with or without NHS. Parasites (5×10^5^, ratio 5∶1, parasites:cells) were incubated with Vero cells (1.0×10^5^) in 50% NHS for 3 hours at 37°C. As controls, parasites and cells (ratio 5∶1) were incubated without NHS. Intracellular parasites in at least 500 cells were quantified. * *p*<0.05; * *p*<0.001.

Firstly, kinetics of invasion with CL Brener, Silvio X10/6, Gamba 05, 860, 812 and R4 strains in Vero cells were determined using a ratio of 5 parasites to each cell. After 30 minutes incubation we detected invasion with all strains analysed (Figure 4A). The highest invasion rate was detected for the strain Cl Brener, 26 parasites per 100 cells after a 120 minute incubation, whereas the strains Silvio X10/6, R4 and Gamba 05 had similar rates of invasion, 12, 14 and 15 parasites per 100 cells, respectively (Figure 4A). Lower rates were detected for the strains 860 and 812, 5 and 7 parasites per 100 cells after 120 minutes, respectively (Figure 4A). To determine whether the complement system would affect the parasite invasion, assays were performed by incubating parasites with cells in the presence of 50% non-immune human serum for 60 minutes at 37°C, and the intracellular parasites were quantified. The amount of intracellular parasites decreased in the presence of normal human serum when compared to invasion in the absence of serum for the complement-sensitive strains (Figure 4B), and a slight difference in invasion rates was detected between them. However, the Gamba 05 strain (complement-resistant strain) maintained similar invasion rate in the presence of serum (Figure 4B), indicating that the decrease in cell invasion was caused by complement-mediated lysis. Furthermore, the invasion rates increased for all strains when the ratios of parasites per cell were increased from 5 to 10 parasites to each cell (Figure 4B); suggesting that the parasite load may affect the success of invasion in the presence of serum. To certify that the complement was limiting the parasite invasion, the assays were performed incubating the parasites and cells for a long time period (3 hours). The longer incubation led to an increase in cell invasion in the absence of serum (Figure 4C); however, in the presence of serum, the rates of invasion of the complement-sensitive strains did no increase significantly. In contrast, the rate of invasion of the strains Gamba 05 (complement-resistant strain) remained similar to the rate of invasion in the absence of serum (Figure 4C). These results indicate that the complement system limit the invasion of complement-sensitive strains likely through the complement-mediated lysis. Importantly, intracellular amastigotes and cell culture-derived trypomastigotes were detected (in the supernatant) after 72 hours of infection in the presence of serum, indicating that parasites that succeeded in infecting the cells were able to progress in their life cycle (not shown).

## Discussion

The host innate immune system is the major barrier for pathogens to succeed in infection, and the complement system is one of the main arms of host defense [9]. Several molecules have been reported to control the complement activation and killing in *T. cruzi* [24], [25], [26], [27]. However, little work has focused on the interactions between insect-transmitted metacyclic trypomastigotes and the complement system in non-immune serum; in addition, whether the complement system can effectively control *T. cruzi* infection.

Kinetic studies of complement activation by metacyclic trypomastigotes have not been reported, and we decided to study several strains from different origins to understand this mechanism. Here, we show evidence that the complement system can kill *T. cruzi* metacyclic trypomastigotes limiting the parasite to succeed in invading the host cells.

Firstly, we showed that the complement activating molecules C1q, C3, MBL, L- and H-ficolins recognize the complement-sensitive epimastigotes. Through the deposition of C3b and C4b, after parasite incubation with normal human serum or serum depleted of complement factors, we showed that the lectin and alternative pathways are the main activators of the complement system by *T. cruzi* in non-immune serum. The role of the alternative pathway to recognize and control pathogen infection, including trypanosomatids, has been extensively explored [17], [18], [35], [36]. However, the lectin pathway has only recently emerged as an important host defense mechanism due to pattern associated recognition molecules, such as MBLs and ficolins, which can recognize a broad range of carbohydrates on invading pathogens [37]. Also, mutations in lectin pathway components have been shown to increase the susceptibility to infection by several microbes [38], [39], [40], [41], [42]. We have recently shown that *T. cruzi* activate the complement lectin pathway [19], and the current results confirm that *T. cruzi* strains isolated from different hosts also activate this pathway. Other pathogens, such as *Leishmania brasiliensis, Salmonella typhimurium* and *Nisseria gonorrheae*, have been shown to activate the lectin pathway [43], [44], strengthening the importance of studying the mechanisms that these pathogens evolved to inhibit this activation and complement killing. On the other hand, as shown here and elsewhere [17], [18], [19], [45] the classical pathway has a limited involvement in the complement activation by *T. cruzi* in non-immune serum, suggesting that natural antibodies would have a limited capacity to recognize *T. cruzi* and activate the complement system.

Assays of complement-mediated lysis, and deposition of C3b and C4b, demonstrated that *T. cruzi* metacyclic trypomastigotes activate the complement system. Furthermore, we have found that some *T. cruzi* strains are highly susceptible to lysis in non-immune serum, whereas other strains differ in their capacities to resist complement killing. Other trypanosomatids, such as *Leishmania sp.* and *Chritidia sp.*, have been shown to be highly susceptible to complement-mediated killing in human serum [36], [46], [47], indicating that the complement system can act as a physiological barrier for trypanosome infection. Since they must have evolved a strategy to evade the complement attack; it appears that, for some pathogens, this strategy actually uses host/vector factors. For example, phagocytosis by macrophages and neutrophils during this period may help them to escape the complement-mediated killing [12], [36], [48]. Furthermore, it has been reported that the saliva and intestinal content of the insect vectors contain complement inhibitors [49], which could also help the parasites to escape the lysis. Although complement regulators could be contributing to complement evasion, it is possible that other unknown evasion strategies are displayed *in vivo*; or perhaps the complement sensitive strains are being transmitted by other routes of infection, such as orally. It is important to consider that the mechanisms employed by *T. cruzi* strains to escape the killing could have evolved differently according to the hosts. Complement system activity is different among humans, opossums, rodents and chickens [50], [51], [52], [53], and their effectiveness in eliminating the parasites could be different. On the other hand, the expression and functionality of complement regulators on *T. cruzi* surface may be different in the strains, resulting in their different abilities to resist complement killing. The expression analysis of complement regulators in resistant and susceptible *T. cruzi* strains could open the possibility to understand this difference.

We have also investigated how the complement system affects the invasion of eukaryotic cells by *T. cruzi*. Experimental invasion *in vitro* in the presence of non-immune human serum at nearly physiological conditions showed that the complement system can limit, but not avoid parasite invasion. Some strains invaded the host cells as soon as 15 minutes, and the invasion increased according with time. Furthermore, in the presence of human serum the levels of invasion of the complement-sensitive strains were dramatically decreased. However, the human serum did not affect the complement-resistant strains to invade the cells. These results suggest that, for the complement system to control the infection, complete clearance during the first minutes of infection is necessary to stop parasites from invading host cells; otherwise, resistant strains can invade the cells and progress in their life cycle. *T. cruzi* metacyclic trypomastigotes differ in their capacity to invade the host cells. There are high and low invasive strains [31], [54], which has been related to the presence of different parasite surface molecules [31]. It is possible that a long parasite exposure to the complement system, before the cell invasion, can affect the success of the infection according to the capacity of the strain to resist the lysis.

Our data support the idea that resistance to complement-mediated killing is not a strict characteristic of the metacyclic trypomastigote stage of *T. cruzi*; rather, there are strains sensitive and resistant to complement killing by human serum. The features that make some strains resistant and others sensitive are still unknown. However, the understanding that *T. cruzi* metacyclic trypomastigotes can be lysed by the complement system or the parasites could express transiently complement regulators facilitating them to resist the lytic effect of the complement and produce infection; open new possibilities to investigate parasite extrinsic mechanisms involved in immune evasion.

A detailed study of the mechanisms of complement system activation and host cell invasion on a molecular level is essential to understand the disease and the development of new therapies. Drug screening initiatives have been focused mainly on parasite metabolism targets rather than on immune evasion strategies [55]. Nevertheless, recent discoveries of complement receptors and immune evasion strategies may reveal promising targets for therapeutic intervention [18], [19], [24], [26], [56], which could be used to control Chagas disease.

In summary, this study demonstrates that the human complement system can kill metacyclic trypomastigotes of *T. cruzi*, and the ability to resist the complement system varies between the strains. Indeed, the complement system acts as a physiological barrier during the infection which parasites have to evade to successfully infect the host.

## Methods

### Parasites and cells

*T. cruzi* epimastigotes were cultivated in liver infusion tryptose medium (bovine liver infusion at 5 g/l, tryptose at 5 g/l, NaCl at 4 g/l, KCl at 0.4 g/l, Na_2_HPO_4_ at 8 g/l, glucose at 2 g/l; pH 7.2), supplemented with haemin at 10 mg/l and fetal bovine serum 10%, at 27°C. Metacyclic trypomastigote forms were obtained as previously described [57]. Vero cells were grown in RPMI 1640 supplemented with 10% FBS at 37°C and 5% CO_2_.

### Complement-mediated killing assay

Normal human serum was obtained from healthy voluntary donors and pooled. Aliquots were stored at −80°C until use. For complement assays, *T. cruzi* metacyclic trypomastigotes (5.0×10^5^) in 100 µl of veronal buffer (VB, 10 mM barbital/NaCl 145 mM/CaCl_2_ 0.15 mM/MgCl_2_ 0.5 mM, pH 7.4) were incubated with 100 µl of normal human serum (NHS) for 5, 10, 30, 60 and 120 minutes at 37°C. Reactions were stopped by the addition of 800 µl of ice-cold VB. Parasite survivors were quantified in a Neubauer chamber by light microscopy.

### Depletion of MBLs and L-ficolins from NHS

Depletion of MBL and L-ficolin from human serum was performed as previously described [19]. Firstly, to deplete NHS of MBL, mannan-agarose (SIGMA-Aldrich) resin equilibrated in HEPES buffer (10 mM HEPES/140 mM NaCl buffer containing 0.15 mM CaCl_2_ and 0.5 mM MgCl_2_, pH 7.4) were incubated with NHS for 2 hour at 4°C on ice, the serum was dialysed against 2 litres of HEPES buffer. Afterwards, the same serum (MBL-depleted) was used for L-ficolins depletion. The serum was incubated for 2 hours on ice with N-acetyl-glucosamine-Sepharose resins (SIGMA-Aldrich, prepared according to manufacture's instructions) equilibrated in 10 mM HEPES/640 mM NaCl buffer, pH 7.4 and 5 mM ethylene diamine tetracetic acid (EDTA). The resin was collected by centrifugation and depleted sera (and non-treated serum) were dialyzed against 10 mM HEPES/140 mM NaCl buffer, pH 7.4. After dialysis, MgCl_2_ and CaCl_2_ were added to final concentrations of 0.5 mM and 0.15 mM, respectively. For NHS depletion of C1q, NHS was incubated in non-immune IgG-sepharose resin (SIGMA-Aldrich, prepared according to manufacture's instructions) equilibrated in 10 mM HEPES/140 mM NaCl buffer, pH 7.4 with 5 mM EDTA and incubated for 2 hours on ice. The resin was collected by centrifugation and depleted sera (and non-treated serum) were dialyzed against 10 mM HEPES/140 mM NaCl buffer, pH 7.4. After dialysis, MgCl_2_ and CaCl_2_ were added to final concentrations of 0.5 mM and 0.15 mM, respectively. Serum depletion was confirmed by ELISA.

### ELISA for complement assays

*T. cruzi* epimastigote or metacyclic trypomastigote forms were fixed in 4% paraformaldehyde, and 100 µl (1.0×10^6^) of parasites were incubated in each well of a 96 well ELISA MaxiSorp plate (Nalge Nunc International) overnight at 4°C with coating buffer (0.1M Na_2_CO_3_/0.1M NaHCO_3_, pH 9.5). The wells were washed three times with phosphate buffered saline (PBS, 137 mM NaCl, 2.7 mM KCl, 4.3 mM Na_2_HPO_4_, 1.47 mM KH_2_PO_4_), then blocked with PBS 3% bovine serum albumin (BSA) for 2 hours at RT. For binding of C1q, C3, L- and H-ficolins, 100 µl of 5% NHS (diluted in VB) were added to each well, followed by a 60 minute incubation at 37°C. For MBL binding, 20% NHS was added to each well and incubated for 60 minutes at 37°C. Wells were washed with PBS, then affinity purified polyclonal goat anti-MBL antiserum (1∶100) (Santa Cruz), polyclonal rabbit anti-L-ficolin antiserum (1∶500), anti-H-ficolin antiserum (1∶500), anti-C1q antiserum (1∶500) and anti-C3 antiserum (1∶500, diluted in PBS 3% BSA) (MRC Immunochemistry Unit, Oxford, England) were added. After a 2 hours incubation at RT, the plate was washed as above, and either rabbit anti-goat IgG-HRP conjugate (1∶2000, diluted in PBS 3% BSA) (Kirkegaard and Perry Laboratories) for anti-MBL or goat anti-rabbit IgG-HRP conjugate (Bio-Rad) (1∶2000, diluted in PBS 3% BSA) for others were added and incubated in the wells for 2 hours at RT. The wells were washed as above, and 100 µl of ABTS peroxidase solution (Kirkegaard and Perry Laboratories) was added. Reactions were incubated at RT for 5 to 10 minutes and stopped with the addition of 50 µl of 1% SDS. Absorbances (at 405 nm) were obtained through spectrophotometric measurement. For kinetics of C3 and C4 deposition, wells previously adsorbed with parasites were incubated with either 5% NHS, or 5% MBLs/L-ficolins-depleted serum, or 5% NHS treated with 10 mM EGTA and 7 mM MgCl_2_ for 5 to 60 minutes at 37°C. Afterwards, the wells were washed with PBS, incubated with rabbit polyclonal anti-C3 antiserum (1∶500) (MRC Immunochemistry Unit) or goat polyclonal anti-C4 antibodies (1∶500, diluted in PBS 3% BSA) (Calbiochem) for 2 hours at RT. After that, wells were washed, incubated with goat anti-rabbit IgG-HRP conjugate (1∶2000, diluted in PBS 3% BSA) (Bio-Rad) and rabbit anti-goat IgG-HRP conjugate (Kirkegaard and Perry Laboratories) (1∶2000, diluted in PBS 3% BSA), respectively and incubated for 2 hours at RT. For development, 100 µl of ABTS peroxidase solution (Kirkegaard and Perry Laboratories) were added, incubated at RT for 5 to 10 minutes and stopped with 50 µl of 1% SDS in H_2_O. Absorbances (at 405 nm) were obtained through spectrophotometric measurement.

### Live/dead viability fluorescence assay

*T. cruzi* metacyclic trypomastigotes of the strain 812 (5.0×10^6^ in 250 µl of PBS) were incubated with 250 µl of NHS or 250 µl of PBS (as control) for 30 minutes at 37°C. After that, cells were washed three times in PBS, resuspended in 1 ml of PBS containing 2 µM of calcein-AM and 4 µM of ethidium homodimer-1 (Live/Dead Viability/Cytotoxity kit, Molecular Probes) and incubated for 30 minutes at RT. Afterwards, cells were centrifuged for 5 minutes at 5,000 rpm, resuspended in 10 µl of PBS, and mounted on slides with Vectashield mounting medium (Vector Laboratories). For fluorescence microscopy an Olympus IX81 inverted microscope, equipped with a U-CMAD3 camera, was used (Olympus).

### Invasion assays

Vero cells from logarithmically grown culture were treated with trypsin, washed once with PBS and seeded on 13 mm cover slips in 24 well plates (1.0×10^5^ cells per well). After 24 h, cells were washed with serum-free RPMI 1640 and incubated with 5.0×10^5^ *T. cruzi* metacyclic trypomastigote forms for 15, 30, 60 and 120 minutes at 37°C with 5% CO_2_. Cells were then washed with PBS, fixed with absolute methanol (MERCK) for 5 minutes, washed with H_2_O and stained with Giemsa for 1 h at RT. Then, they were washed with H_2_O and slides were mounted with aqueous mounting medium (Biomeda). Intracellular parasites were quantified by light microscopy, counting at least 500 cells per slide. For invasion in the presence of NHS, cells were seeded as described above and metacyclic trypomastigotes 5.0×10^5^ (ratio 5∶1, parasites:cells) and 1.0×10^6^ (ratio 10∶1, parasites:cells) were added in the presence of 50% NHS (diluted in serum-free RPMI 1640, in a final volume of 500 µl), followed by 60 or 180 minutes incubation at 37°C with 5% CO_2_. As controls, 5.0×10^5^ parasites were added to wells containing Vero cells without NHS. Staining and quantification were performed as described above.

### Data presentation and statistical analysis

All data shown are the average of two or three experiments performed at least in triplicate. All data are shown as mean +/− SD. Comparisons among groups were made by the unpaired t-test for repeated measures using GraphPad Prism version 5.00 for Windows, GraphPad Software, San Diego California USA. Values of *p*<0.05 with confidence interval of 90% were considered statistically significant unless otherwise specified.

## Supporting Information

Figure S1 ELISA to analyse human serum depleted of MBL/L-ficolins and C1q. A) 100 µl of 5% normal human serum, 5% C1q-depleted serum and 5% MBL/L-ficolins-depleted serum were incubated with *T. cruzi* epimastigotes of the strain X10/6 (previously adsorbed in ELISA plates) for 1 hour at 37°C. Polyclonal antibodies anti-C1q was used for C1q detection. Reactions were developed with ABTS peroxidase solution and absorbances obtained at 405 nm. NHS (normal human serum), MBL/L-fic-dep (MBL and L-ficolins-depleted serum), C1q-dep (C1q-depleted serum). B) Similar to A, except that polyclonal antibodies anti-MBL was used for detection. C) Similar to A, except that polyclonal antibodies anti-L-ficolins was used for detection. D) Similar to A, except that polyclonal antibodies anti-C3 was used for detection. Significance is shown on each graph. Data from depleted serum was compared to normal human serum.
(2.26 MB TIF)

Figure S2 Morphological features of *T. cruzi* epimastigote and metacyclic trypomastigote stage. A) *T. cruzi* strain Silvio X10/6 stained with Giensa. 1 and 2, epimastigotes (3 days of culture); 3 and 4, metacyclic trypomastigotes. Metacyclic trypomastigotes were purified by ion exchange chromatography (in DEAE-cellulose). The efficiency of the purification was 97.5%. B) Western blotting to detect the expression of gp82 (metacyclic trypomastigotes stage-specific protein). Protein extract from *T. cruzi* strain Silvio X10/6 (1.0×107/well) epimastigotes and metacyclic trypomastigotes were obtained with Triton-X100 1% in PBS and separated by SDS/PAGE. The proteins were transferred to nitrocellulose membranes and blotted with monoclonal antibodies anti-3F6 (which recognizes gp82). D) *T. cruzi* strain Gamba 05 stained with Giensa. 1 and 2, epimastigotes (4 days of culture); 3 and 4, metacyclic trypomastigotes. Metacyclic trypomastigotes were obtained as described in A. The efficiency of the purification was 99%. C) Western blotting to detect the expression of the metacyclic trypomastigotes stage-specific protein gp82 in the strain Gamba 05. The procedure was as described in B, except that 2.0×107 parasites/well was used. E, epimastigotes; M, metacyclic trypomastigotes.
(5.99 MB TIF)

Figure S3 Serum lysis of *T. cruzi* metacyclic trypomastigotes is dependent on the complement system. Lysis of *T. cruzi* metacyclic trypomastigotes with human serum is inhibited by serum treatment with ethylenediamine tetraacetic acid (EDTA), protease inhibitors or using complement consumed serum. A) Metacyclic trypomastigotes (5.0×105) of the strain Silvio X10/6 were incubated for 1 hour at 37°C with normal human serum (NHS), normal human serum treated with 10 mM EDTA (NHS+EDTA), normal human serum treated with protease inhibitors (NHS+P Inhibitors; 1 mM phenylmethylsulfonyl fluoride, 1 µM aprotinin and 50 pM soybean trypsin inhibitor, added just before the complement lysis assay) or complement consumed normal human serum (NHS Consumed; consumption of complement were obtained by incubating the serum at 37°C for 2 hours with protein extract of 5.0×106 *T. cruzi* epimastigotes). Parasite survivors were quantified. B) Similar to A, but with the strain 860. Significance is shown on each graph. Data from NHS treatment were compared to the values from NHS-EDTA, NHS-PI and NHS Cons separately using unpaired t-test.
(0.38 MB TIF)

## References

[1] Teixeira AR, Nitz N, Guimaro MC, Gomes C, Santos-Buch CA (2006). Chagas disease.. Postgrad Med J.

[2] Gurtler RE, Ceballos LA, Ordonez-Krasnowski P, Lanati LA, Stariolo R (2009). Strong Host-Feeding Preferences of the Vector Triatoma infestans Modified by Vector Density: Implications for the Epidemiology of Chagas Disease.. PLoS Negl Trop Dis.

[3] Munoz J, Coll O, Juncosa T, Verges M, del Pino M (2009). Prevalence and vertical transmission of Trypanosoma cruzi infection among pregnant Latin American women attending 2 maternity clinics in Barcelona, Spain.. Clin Infect Dis.

[4] Piron M, Verges M, Munoz J, Casamitjana N, Sanz S (2008). Seroprevalence of Trypanosoma cruzi infection in at-risk blood donors in Catalonia (Spain).. Transfusion.

[5] Sanchez Negrette O, Mora MC, Basombrio MA (2005). High prevalence of congenital Trypanosoma cruzi infection and family clustering in Salta, Argentina.. Pediatrics.

[6] Valente SA, da Costa Valente V, das Neves Pinto AY, de Jesus Barbosa Cesar M, dos Santos MP (2009). Analysis of an acute Chagas disease outbreak in the Brazilian Amazon: human cases, triatomines, reservoir mammals and parasites.. Trans R Soc Trop Med Hyg.

[7] Yoshida N (2008). Trypanosoma cruzi infection by oral route: how the interplay between parasite and host components modulates infectivity.. Parasitol Int.

[8] Buscaglia CA, Campo VA, Frasch AC, Di Noia JM (2006). Trypanosoma cruzi surface mucins: host-dependent coat diversity.. Nat Rev Microbiol.

[9] Lambris JD, Ricklin D, Geisbrecht BV (2008). Complement evasion by human pathogens.. Nat Rev Microbiol.

[10] Kollien AH, Schaub GA (1998). Development of Trypanosoma cruzi after starvation and feeding of the vector - a review.. Tokai J Exp Clin Med.

[11] Kollien AH, Schaub GA (2000). The development of Trypanosoma cruzi in triatominae.. Parasitol Today.

[12] Bijovsky AT, Milder RV, Abrahamsohn IA, Sinhorini IL, Mariano M (1984). The influence of lymphatic drainage in experimental Trypanosoma cruzi infection.. Acta Trop.

[13] Schuster JP, Schaub GA (2000). Trypanosoma cruzi: skin-penetration kinetics of vector-derived metacyclic trypomastigotes.. Int J Parasitol.

[14] Leon-Perez F, Gomez-Garcia L, Alejandre-Aguilar R, Lopez R, Monteon VM (2007). Mexican Trypanosoma cruzi isolates: in vitro susceptibility of epimastigotes to anti-trypanosoma cruzi drugs and metacyclic forms to complement-mediated lysis.. Vector Borne Zoonotic Dis.

[15] Yoshida N, Araguth MF (1987). Trypanolytic activity and antibodies to metacyclic trypomastigotes of Trypanosoma cruzi in non-Chagasic human sera.. Parasite Immunol.

[16] Joiner K, Hieny S, Kirchhoff LV, Sher A (1985). gp72, the 72 kilodalton glycoprotein, is the membrane acceptor site for C3 on Trypanosoma cruzi epimastigotes.. J Exp Med.

[17] Krettli AU, Pontes de Carvalho LC (1985). Binding of C3 fragments to the Trypanosoma cruzi surface in the absence of specific antibodies and without activation of the complement cascade.. Clin Exp Immunol.

[18] Joiner K, Sher A, Gaither T, Hammer C (1986). Evasion of alternative complement pathway by Trypanosoma cruzi results from inefficient binding of factor B.. Proc Natl Acad Sci U S A.

[19] Cestari I, Krarup A, Sim RB, Inal JM, Ramirez MI (2009). Role of early lectin pathway activation in the complement-mediated killing of Trypanosoma cruzi.. Mol Immunol.

[20] Krettli AU, Weisz-Carrington P, Nussenzweig RS (1979). Membrane-bound antibodies to bloodstream Trypanosoma cruzi in mice: strain differences in susceptibility to complement-mediated lysis.. Clin Exp Immunol.

[21] Kipnis TL, Tambourgi DV, Alves MJ, Silva WD (1992). Comparison of the C-mediating killing activity and C-activating properties of mouse monoclonal and polyclonal antibodies against Trypanosoma cruzi.. Mediators Inflamm.

[22] Almeida IC, Milani SR, Gorin PA, Travassos LR (1991). Complement-mediated lysis of Trypanosoma cruzi trypomastigotes by human anti-alpha-galactosyl antibodies.. J Immunol.

[23] Almeida IC, Ferguson MA, Schenkman S, Travassos LR (1994). Lytic anti-alpha-galactosyl antibodies from patients with chronic Chagas' disease recognize novel O-linked oligosaccharides on mucin-like glycosyl-phosphatidylinositol-anchored glycoproteins of Trypanosoma cruzi.. Biochem J.

[24] Cestari I, Evans-Osses I, Freitas JC, Inal JM, Ramirez MI (2008). Complement C2 receptor inhibitor trispanning confers an increased ability to resist complement-mediated lysis in Trypanosoma cruzi.. J Infect Dis.

[25] Atayde VD, Neira I, Cortez M, Ferreira D, Freymuller E (2004). Molecular basis of non-virulence of Trypanosoma cruzi clone CL-14.. Int J Parasitol.

[26] Norris KA (1998). Stable transfection of Trypanosoma cruzi epimastigotes with the trypomastigote-specific complement regulatory protein cDNA confers complement resistance.. Infect Immun.

[27] Tambourgi DV, Kipnis TL, da Silva WD, Joiner KA, Sher A (1993). A partial cDNA clone of trypomastigote decay-accelerating factor (T-DAF), a developmentally regulated complement inhibitor of Trypanosoma cruzi, has genetic and functional similarities to the human complement inhibitor DAF.. Infect Immun.

[28] Roellig DM, Ellis AE, Yabsley MJ (2009). Oral transmission of Trypanosoma cruzi with opposing evidence for the theory of carnivory.. J Parasitol.

[29] Bahia MT, Tafuri WL, Caliari MV, Veloso VM, Carneiro CM (2002). Comparison of Trypanosoma cruzi infection in dogs inoculated with blood or metacyclic trypomastigotes of Berenice-62 and Berenice-78 strains via intraperitoneal and conjunctival routes.. Rev Soc Bras Med Trop.

[30] Roellig DM, Ellis AE, Yabsley MJ (2009). Genetically different isolates of Trypanosoma cruzi elicit different infection dynamics in raccoons (Procyon lotor) and Virginia opossums (Didelphis virginiana).. Int J Parasitol.

[31] Yoshida N (1983). Surface antigens of metacyclic trypomastigotes of Trypanosoma cruzi.. Infect Immun.

[32] Samanovic M, Molina-Portela MP, Chessler A-DC, Burleigh BA, Raper J (2009). Trypanosome Lytic Factor, an Antimicrobial High-Density Lipoprotein, Ameliorates *Leishmania* infection.. PLoS Pathogens.

[33] Perez-Morga D, Vanhollebeke B, Paturiaux-Hanocq F, Nolan DP, Lins L (2005). Apolipoprotein L-I promotes trypanosome lysis by forming pores in lysosomal membranes.. Science.

[34] Pays E, Vanhollebeke B, Vanhamme L, Paturiaux-Hanocq F, Nolan DP (2006). The trypanolytic factor of human serum.. Nat Rev Microbiol.

[35] Kipnis TL, Krettli AU, Dias da Silva W (1985). Transformation of trypomastigote forms of Trypanosoma cruzi into activators of alternative complement pathway by immune IgG fragments.. Scand J Immunol.

[36] Dominguez M, Moreno I, Lopez-Trascasa M, Torano A (2002). Complement interaction with trypanosomatid promastigotes in normal human serum.. J Exp Med.

[37] Garlatti V, Belloy N, Martin L, Lacroix M, Matsushita M (2007). Structural insights into the innate immune recognition specificities of L- and H-ficolins.. Embo J.

[38] Turner MW, Hamvas RM (2000). Mannose-binding lectin: structure, function, genetics and disease associations.. Rev Immunogenet.

[39] Thiel S, Frederiksen PD, Jensenius JC (2006). Clinical manifestations of mannan-binding lectin deficiency.. Mol Immunol.

[40] Stengaard-Pedersen K, Thiel S, Gadjeva M, Moller-Kristensen M, Sorensen R (2003). Inherited deficiency of mannan-binding lectin-associated serine protease 2.. N Engl J Med.

[41] Rantala A, Lajunen T, Juvonen R, Bloigu A, Silvennoinen-Kassinen S (2008). Mannose-binding lectin concentrations, MBL2 polymorphisms, and susceptibility to respiratory tract infections in young men.. J Infect Dis.

[42] Ivanova M, Ruiqing J, Matsushita M, Ogawa T, Kawai S (2008). MBL2 single nucleotide polymorphism diversity among four ethnic groups as revealed by a bead-based liquid array profiling.. Hum Immunol.

[43] Devyatyarova-Johnson M, Rees IH, Robertson BD, Turner MW, Klein NJ (2000). The lipopolysaccharide structures of Salmonella enterica serovar Typhimurium and Neisseria gonorrhoeae determine the attachment of human mannose-binding lectin to intact organisms.. Infect Immun.

[44] Ambrosio AR, De Messias-Reason IJ (2005). Leishmania (Viannia) braziliensis: interaction of mannose-binding lectin with surface glycoconjugates and complement activation. An antibody-independent defence mechanism.. Parasite Immunol.

[45] Barrett VJ, Leiby DA, Odom JL, Otani MM, Rowe JD (1997). Negligible prevalence of antibodies against Trypanosoma cruzi among blood donors in the southeastern United States.. Am J Clin Pathol.

[46] Moreno I, Molina R, Torano A, Laurin E, Garcia E (2007). Comparative real-time kinetic analysis of human complement killing of Leishmania infantum promastigotes derived from axenic culture or from Phlebotomus perniciosus.. Microbes Infect.

[47] Pearson RD, Steigbigel RT (1980). Mechanism of lethal effect of human serum upon Leishmania donovani.. J Immunol.

[48] Peters NC, Sacks DL (2009). The impact of vector-mediated neutrophil recruitment on cutaneous leishmaniasis.. Cell Microbiol.

[49] Barros VC, Assumpção JG, Cadete AM, Santos VC, Cavalcante RR (2009). The role of salivary and intestinal complement system inhibitors in the midgut protection of triatomines and mosquitoes.. PLoS ONE.

[50] Wirtz GH, Westfall SA (1967). Immune complement of the opossum.. Immunochemistry.

[51] Parmentier HK, Baelmans R, Savelkoul HF, Dorny P, Demey F (2004). Serum haemolytic complement activities in 11 different MHC (B) typed chicken lines.. Vet Immunol Immunopathol.

[52] Koene RA, McKenzie IF (1973). A comparison of the cytolytic action of guinea pig and rabbit complement on sensitized nucleated mouse cells.. J Immunol.

[53] Ish C, Ong GL, Desai N, Mattes MJ (1993). The specificity of alternative complement pathway-mediated lysis of erythrocytes: a survey of complement and target cells from 25 species.. Scand J Immunol.

[54] Yoshida N (2006). Molecular basis of mammalian cell invasion by Trypanosoma cruzi.. An Acad Bras Cienc.

[55] Decastro SL (1993). The challenge of Chagas' disease chemotherapy: an update of drugs assayed against Trypanosoma cruzi (a review).. Acta Trop.

[56] Ferreira V, Valck C, Sanchez G, Gingras A, Tzima S (2004). The classical activation pathway of the human complement system is specifically inhibited by calreticulin from Trypanosoma cruzi.. J Immunol.

[57] Contreras VT, Salles JM, Thomas N, Morel CM, Goldenberg S (1985). In vitro differentiation of Trypanosoma cruzi under chemically defined conditions.. Mol Biochem Parasitol.

