# Supplementary figures and images for: Inefficient Complement System Clearance of Trypanosoma cruzi Metacyclic Trypomastigotes Enables Resistant Strains to Invade Eukaryotic Cells
Source: PLoS One. 2010 Mar 16;5(3):e9721. doi: 10.1371/journal.pone.0009721 (PMC2838796; doi:10.1371/journal.pone.0009721)

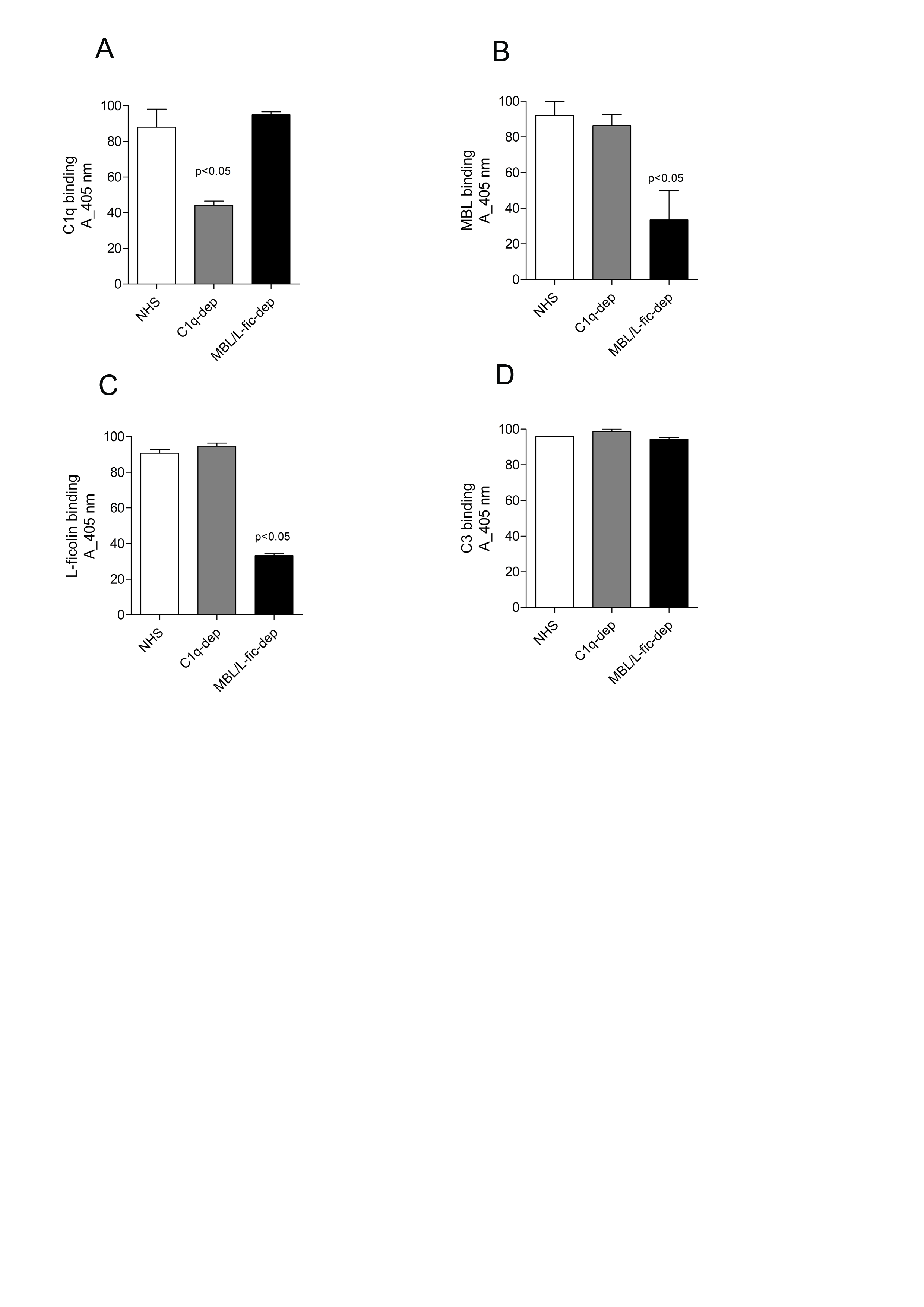

Supplement: Figure S1 — ELISA to analyse human serum depleted of MBL/L-ficolins and C1q. A) 100 µl of 5% normal human serum, 5% C1q-depleted serum and 5% MBL/L-ficolins-depleted serum were incubated with T. cruzi epimastigotes of the strain X10/6 (previously adsorbed in ELISA plates) for 1 hour at 37°C. Polyclonal antibodies anti-C1q was used for C1q detection. Reactions were developed with ABTS peroxidase solution and absorbances obtained at 405 nm. NHS (normal human serum), MBL/L-fic-dep (MBL and L-ficolins-depleted serum), C1q-dep (C1q-depleted serum). B) Similar to A, except that polyclonal antibodies anti-MBL was used for detection. C) Similar to A, except that polyclonal antibodies anti-L-ficolins was used for detection. D) Similar to A, except that polyclonal antibodies anti-C3 was used for detection. Significance is shown on each graph. Data from depleted serum was compared to normal human serum. (2.26 MB TIF) [file pone.0009721.s001.tif]

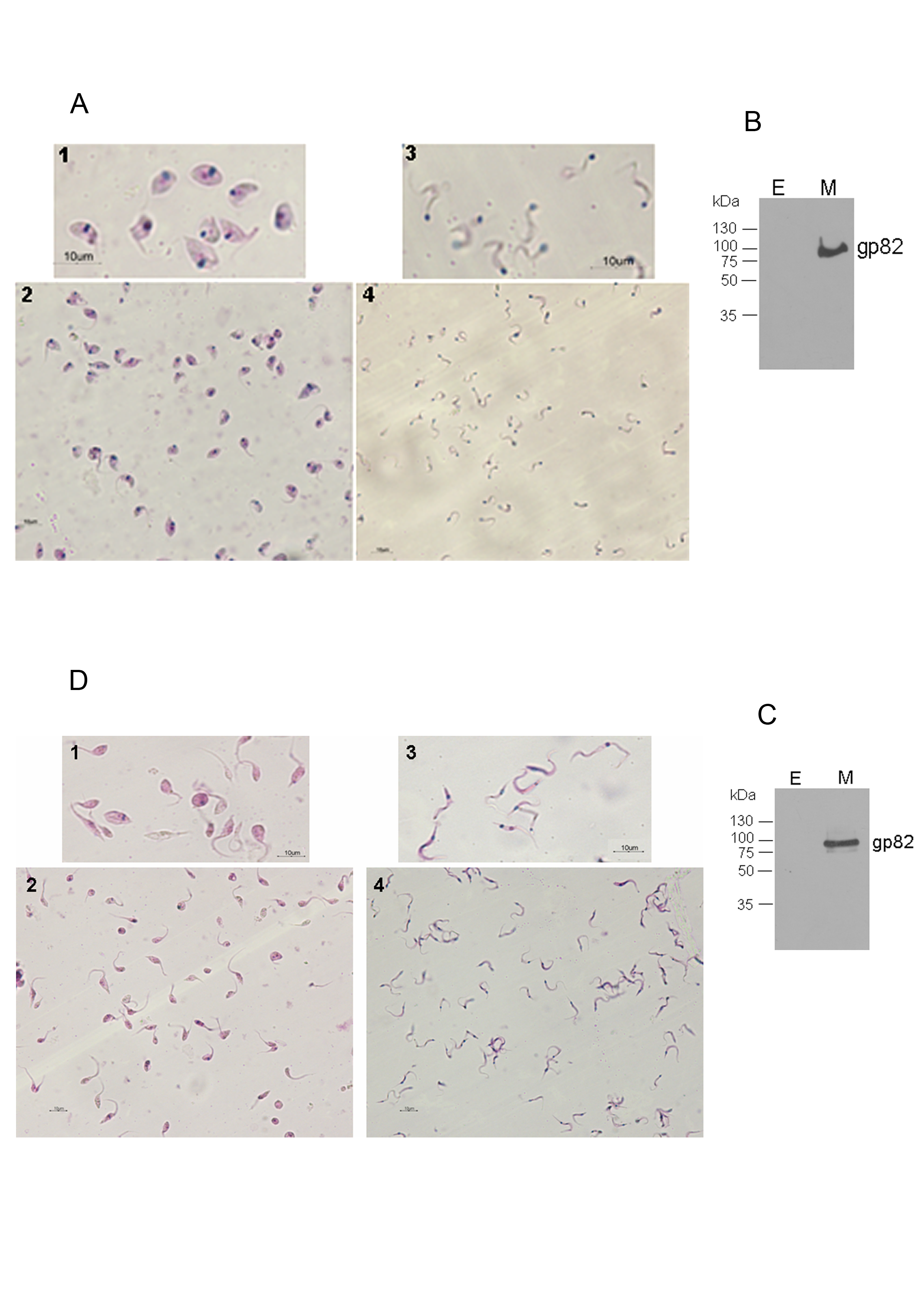

Supplement: Figure S2 — Morphological features of T. cruzi epimastigote and metacyclic trypomastigote stage. A) T. cruzi strain Silvio X10/6 stained with Giensa. 1 and 2, epimastigotes (3 days of culture); 3 and 4, metacyclic trypomastigotes. Metacyclic trypomastigotes were purified by ion exchange chromatography (in DEAE-cellulose). The efficiency of the purification was 97.5%. B) Western blotting to detect the expression of gp82 (metacyclic trypomastigotes stage-specific protein). Protein extract from T. cruzi strain Silvio X10/6 (1.0×10^7^/well) epimastigotes and metacyclic trypomastigotes were obtained with Triton-X100 1% in PBS and separated by SDS/PAGE. The proteins were transferred to nitrocellulose membranes and blotted with monoclonal antibodies anti-3F6 (which recognizes gp82). D) T. cruzi strain Gamba 05 stained with Giensa. 1 and 2, epimastigotes (4 days of culture); 3 and 4, metacyclic trypomastigotes. Metacyclic trypomastigotes were obtained as described in A. The efficiency of the purification was 99%. C) Western blotting to detect the expression of the metacyclic trypomastigotes stage-specific protein gp82 in the strain Gamba 05. The procedure was as described in B, except that 2.0×10^7^ parasites/well was used. E, epimastigotes; M, metacyclic trypomastigotes. (5.99 MB TIF) [file pone.0009721.s002.tif]

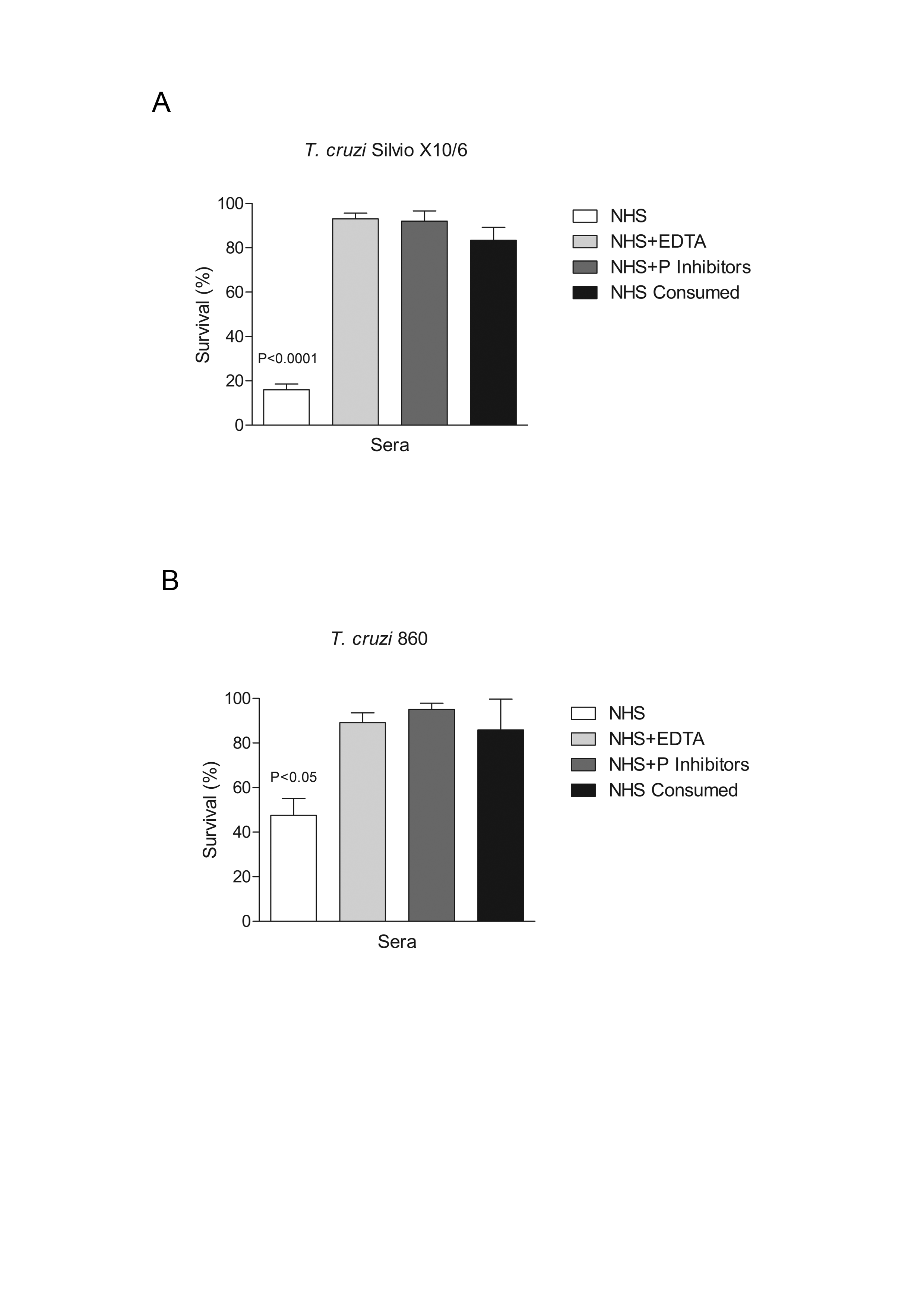

Supplement: Figure S3 — Serum lysis of T. cruzi metacyclic trypomastigotes is dependent on the complement system. Lysis of T. cruzi metacyclic trypomastigotes with human serum is inhibited by serum treatment with ethylenediamine tetraacetic acid (EDTA), protease inhibitors or using complement consumed serum. A) Metacyclic trypomastigotes (5.0×10^5^) of the strain Silvio X10/6 were incubated for 1 hour at 37°C with normal human serum (NHS), normal human serum treated with 10 mM EDTA (NHS+EDTA), normal human serum treated with protease inhibitors (NHS+P Inhibitors; 1 mM phenylmethylsulfonyl fluoride, 1 µM aprotinin and 50 pM soybean trypsin inhibitor, added just before the complement lysis assay) or complement consumed normal human serum (NHS Consumed; consumption of complement were obtained by incubating the serum at 37°C for 2 hours with protein extract of 5.0×10^6^ T. cruzi epimastigotes). Parasite survivors were quantified. B) Similar to A, but with the strain 860. Significance is shown on each graph. Data from NHS treatment were compared to the values from NHS-EDTA, NHS-PI and NHS Cons separately using unpaired t-test. (0.38 MB TIF) [file pone.0009721.s003.tif]
